# “I just wanted money for food”: a qualitative study of the experiences of Australians during the COVID-19 pandemic

**DOI:** 10.1007/s10389-023-01952-y

**Published:** 2023-06-05

**Authors:** Heidi Green, Catherine MacPhail, Ritin Fernandez

**Affiliations:** 1 School of Health and Society, University of Wollongong, Wollongong, NSW Australia; 2 Centre for Evidence Based Initiatives in Health Care: A JBI Centre of Excellence, St George Hospital, Kogarah, NSW Australia; 3 School of Nursing and Midwifery, University of Newcastle, Newcastle, NSW Australia

**Keywords:** Social determinants of health, Housing instability, Food insecurity, Well-being, COVID-19

## Abstract

**Aim:**

The social and economic impacts that have occurred during the COVID-19 pandemic can disproportionally affect those already experiencing poverty or at risk of poverty. Therefore, this study sought to explore the relationship between well-being and social determinants of health among Australian adults during the pandemic.

**Subject and Methods:**

Semi-structured interviews were undertaken with 20 participants, aged 21–65 years, from various socioeconomic areas.

**Results:**

Three main themes emerged from the analysis of the data: food security; housing outcomes; and psychological and emotional impact. Participants in low socioeconomic areas struggled with food security, having to access food banks, which was precipitated by employment loss during the pandemic. Some female participants experienced worsening inequalities and lack of financial and housing stability, affecting their overall well-being.

**Conclusion:**

This study identified that there was a clear social divide between adults living in low socioeconomic areas compared with those living in high socioeconomic areas, with participants in low socioeconomic areas faring worse in terms of exacerbated social determinants of health and consequent impacts on well-being.

## Introduction

The emergence of the infectious disease SARS-CoV-2 in 2019 triggered a global pandemic that has had profound impacts on individuals and communities across the world. In response, public health measures were globally implemented to prevent widespread transmission of the SARS-CoV-2 virus (Burström and Tao 2020). Nationwide lockdowns and social distancing actions were instigated in a majority of countries. Despite lockdowns being an effective public health action to prevent the spread of COVID-19, they can have varying impacts on different populations (Burström and Tao 2020; O’Sullivan et al. 2020). For example, the enforced lockdowns in Australia paused most social and economic activity, with the flow-on effect resulting in substantial loss of employment and income for some population groups (O’Sullivan et al. 2020), while those employed in sectors that allowed working from home had the benefit of limited income and employment loss (Burström and Tao 2020). It is important to highlight that a fundamental risk in any public health crisis is the aggravation of existing health and social inequalities (Abrams and Szefler 2020). In some areas of Australia, such as Melbourne, Victoria, strict lockdowns ensued on six occasions between 2020 and 2021, with more than 260 days spent in lockdown (Australian Bureau of Statistics 2021). A hard lockdown of a public housing tower in Melbourne saw marginalised populations, such as Aboriginal and Torres Strait Islanders and other ethnic minority groups, subject to policing and coercion under the disguise of public health intervention (Silva 2020). New South Wales, the most populous state in Australia, experienced two strict lockdowns, the longest lockdown occurring in 2021 from 26 June – 11 October (Australian Bureau of Statistics 2021). There is a growing body of global quantitative evidence indicating that the experience in Australia is similar to other high-income countries (Turner-Musa et al. 2020; Upshaw et al. 2021) and comparable to both middle- and low-income countries (Figueiredo et al. 2020; Pandey et al. 2021).

While some of the changes that occurred as a response to the pandemic have resulted in population groups losing employment, losing income, experiencing housing instability and losing adequate food supply, the impact of these changes is dependent upon the state of their pre-pandemic social determinants of health (Palmer et al. 2019). Basic human needs, such as housing, food, income, employment, and access to health care, are collectively known as the social determinants of health (Sharma et al. 2021). According to the World Health Organization (WHO), social determinants of health “are the circumstances in which people are born, grow up, live, work and age, and the systems put in place to deal with illness. These circumstances are in turn shaped by a wider set of forces: economics, social policies, and politics” (World Health Organization 2013.) The social determinants of health, housing, and food, have a bi-directional relationship, with vulnerable populations having to spend significant proportions of their income on housing, leading to less money being able to be spent on food (Kirkpatrick and Tarasuk 2011). While some population groups already experienced poor health as a consequence of the social determinants of health prior to the pandemic, the consequences of the COVID-19 pandemic have exacerbated social determinants of health. Consequently, further health inequalities are formed through social positioning and stratification, whereby power and distribution of resources are unequal, creating health differences between population groups and the potential for future inequitable experiences (Choi et al. 2021).

The social and economic impacts that have occurred during the pandemic can disproportionally affect those already experiencing poverty or at risk of poverty, such as those populations residing in low socioeconomic areas (Choi et al. 2021). Exacerbation of the social determinants of health aggravates inequalities and can adversely affect population well-being, particularly during a public health crisis such as a pandemic. The contemporary idea of well-being involves an individual’s physical, emotional, psychological, financial, and spiritual well-being and embraces elements of quality of life such as life satisfaction and fulfilment (Medvedev and Landhuis 2018; Netemeyer et al. 2018). While there is increasing quantitative literature on the impact of the social determinants of health during the COVID-19 pandemic, there is a paucity of qualitative research reflecting the lived experience of these factors. Therefore, this study aims to explore the experiences of Australian adults in differing socioeconomic groups relating to the impacts of the COVID-19 pandemic on social determinants of health and the effects this has had on their well-being.

## Methods

### Design

This descriptive qualitative study is embedded within a sequential mixed-methods study exploring the relationship between well-being and social determinants of health among Australian adults during the COVID-19 pandemic. (Green et al. 2021, 2022) Ethics approval was received from the University of Wollongong Human Research Ethics Committee (HREC), approval no. 2020/306, prior to commencing this study.

### Participants and recruitment

Purposive sampling was used to identify and recruit participants into the study. Participants who completed an online survey as part of the larger national study provided their contact details to participate in an interview and were purposively selected. The type of purposeful sampling used in this study was quota sampling, which enabled the researchers to select participants based on the number of characteristics they possess. A total of 84 participants were contacted for the semi-structured interviews, four refused to participate, and 60 did not respond to the invitation. This strategy was used to recruit a comprehensive cross section of participants from across the Socio-Economic Indexes for Areas (SEIFA), Index of Relative Socio-economic Advantage and Disadvantage (IRSAD) (Australian Bureau of Statistics 2018), and included the characteristics of age, gender, socioeconomic status, and Australian state or territory. It was not a criterion that participants were Australian citizens to participate in the study. The IRSAD is used to gather and collate data regarding the social and economic conditions of individuals by local government area and provides a score based on relative advantage or disadvantage, with a high score indicating greater socioeconomic advantage, and a low score signifying greater disadvantage (Australian Bureau of Statistics 2018). To ensure that contact information remained separate from survey information, each participant that agreed to participate in the semi-structured interviews had a study code applied to their survey responses by an independent researcher. Following the application of the study code, all contact details were exported by the independent researcher into a password-protected excel file. Purposive sampling was conducted by the primary researcher (HG) using the study codes, which were then provided to the independent researcher who gave the corresponding contact details to the primary researcher. During the recruitment process, the participants were contacted via email and provided with additional information regarding the qualitative study and a consent form.

### Data collection

**Fig. 1:**
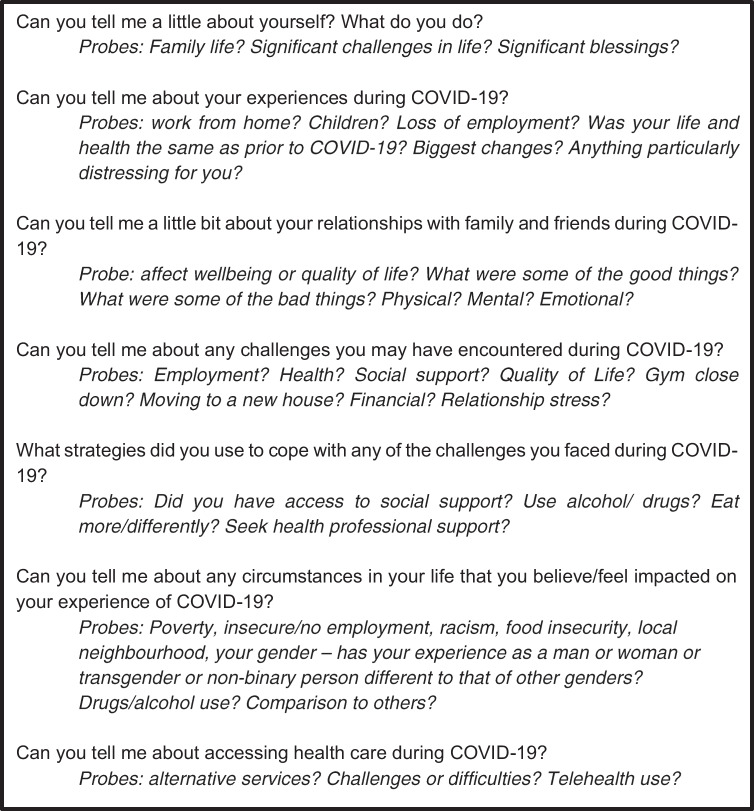
Semi-structured interview questions

As this study aimed to gain a rich insight into adults’ experiences of the social determinants of health during the COVID-19 pandemic, one-on-one semi-structured interviews were the most appropriate data collection method (Braun and Clarke 2006). Semi-structured interviews allow for a deep understanding of the phenomenon being studied, by investigating the “why” of a research question (Fylan 2005). A semi-structured interview guide to broadly explore experiences during COVID-19, circumstances that impacted their experience of COVID-19, coping strategies used during COVID-19, and experiences accessing food and housing was informed by the results of the quantitative analysis, the aim of the study and a review of literature on the social determinants of health. Probing questions were used to generate further explanation from the participants (Fig. 1).

Due to COVID-19 pandemic restrictions and the geographical location of the participants’, one-on-one semi-structured interviews were conducted via either telephone or videoconference (via Zoom) at a mutually agreed time and date between March and August 2021. All interviews were conducted by a female PhD candidate (HG) who is an experienced public health professional and had previous experience in qualitative interviewing. Prior to commencing the interviews, study details including the study outline and aims that had been emailed to the participants during recruitment were discussed, and participants were assured that the interview was voluntary and that they could withdraw their consent at any time. All participants provided signed informed consent prior to the commencement of the interview; this included consent for audio-recording of the interview. Each one-on-one semi-structured interview was digitally audio-recorded, with participants assigned a unique pseudonym following the interview to ensure anonymity. The semi-structured interviews ranged from 30 to 60 minutes. Participants were provided with a $50 shopping gift card for their time. A total of 20 participants were interviewed, with data saturation, the point at which no new information is yielded (Braun and Clarke 2021), thought to be achieved at 17 interviews; however, three more interviews were conducted as confirmation that data saturation had occurred.

### Data analysis

All interview audio recordings were transcribed verbatim using a professional transcription service. Data analysis was supported by NVivo version 12 (QSR International Pty Ltd 2018) with semi-structured interview transcripts imported into this software. All transcripts were checked for accuracy against the audio recordings by the first author. All data were coded initially by the first author and checked by the research team. Discrepancies in the codes generated were resolved with discussion among the research team. The data collected from the semi-structured interviews were analysed using an inductive thematic analysis approach as described by Braun and Clarke (2006). An inductive thematic approach allows for meaning to be derived from the content of the data rather than the researchers’ preconceived ideas and notions. However, it also included some elements of a deductive approach by using the results from the online survey. Inductive thematic analysis was conducted through fundamental phases: immersion within the data, generation of initial codes and themes, clarifying that the codes were logical and supported by the data, defining the themes, and developing sub-themes and reviewing the themes for quality (Braun and Clarke 2006).

### Rigour

The rigour of this research, including trustworthiness and quality, was enhanced by using the four components of credibility, transferability, dependability and confirmability described by Lincoln and Guba (1985). Credibility was achieved by ensuring that participants were a diverse sample; that is, from a range of geographical locations and socioeconomic areas. Additionally, we ensured that data saturation had occurred within each of the geographic locations, rather than only across the sample as a whole. Transferability was enhanced by ensuring the participants were geographically dispersed across Australia and from various socioeconomic groups, as well as through the use of detailed descriptions of participants’ circumstances and experiences. In the context of this study, dependability was achieved by systematic documentation of the interpretation of the transcripts and theming. Lastly, confirmability was established through ongoing reflexivity and ensuring the interpretation of the data was representative of the participants' quotes.

## Results

**Fig. 2:**
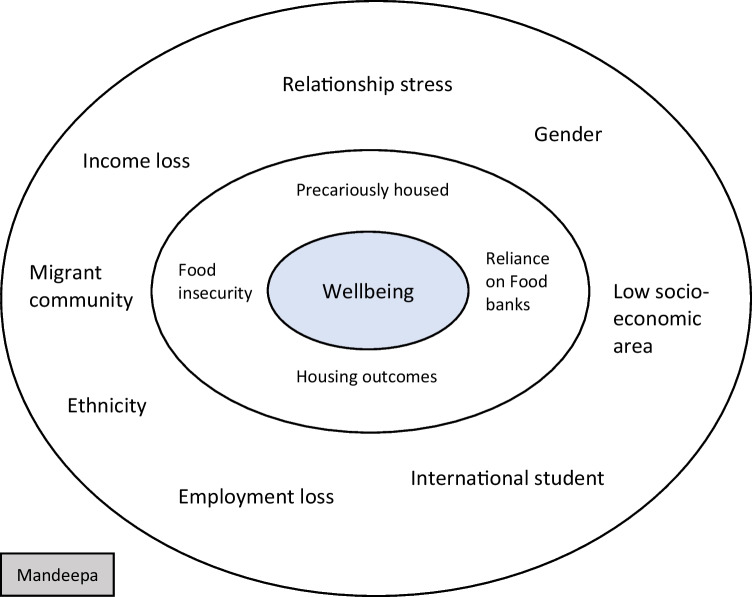
Interplay of social determinants of health and well-being

Twenty people (10 female, 8 male, 1 non-binary and 1 transgender) were recruited from various socioeconomic areas in all states and territories throughout Australia. Participants ranged in age from 21 years to 65 years (Table 1). Three main themes emerged from the analysis of the data: food-related concerns; housing outcomes; and psychological and emotional impact. These themes are described in further detail below, with verbatim quotes from the participants to illustrate key themes. Quotes were selected based on best representation of the themes overall and where the experiences contrasted with the main thematic ideas (Saldaña 2021). Figure 2 details a case study of one of the participants, demonstrating the interplay of the social determinants of health and well-being. The centre ring in the figure is the participant’s well-being. The inner ring displays the leading social determinants of health, food and housing, that the participant experienced during the pandemic influencing her well-being. These are also the main themes of this paper. The outer ring shows all the other existing social determinants of health experienced by the participant, which are interconnected and affecting her well-being during the pandemic. In keeping with the interconnected nature of social determinants (Jonsson et al. 2018), the social determinants of health within the outer ring impact on those social determinants of health in the inner ring.

**Table 1:** Characteristics of participants

Pseudonym	Age (years)	Gender	Socioeconomic status
Aaron	65	Male	High
Alicia	31	Female	High
Clara	38	Female	High
Dominic	55	Male	Low
Emma	31	Female	High
Haimi	25	Female	Low
Joshua	43	Male	Low
Jayda	46	Female	Low
Kailani	26	Female	Low
Karlee	24	Female	Low
Mandeepa	26	Female	Low
Manaia	52	Female	Low
Marcel	51	Male	High
Nick	52	Male	High
Nyah	46	Transgender	Low
Parrie	64	Male	High
Reuben	61	Male	Low
Sergio	35	Male	High
Trey	40	Non-binary	High
Xiuying	21	Female	High

## Food security

For most participants who resided in low socioeconomic areas, accessing food during the COVID-19 pandemic was described as stressful and challenging, especially when compared to those who resided in high socioeconomic areas. Being able to access food was often reported as difficult with limited financial capacity, frequently precipitated through loss of employment or reduced working hours as part of casual employment. Participants noted this struggle by stating “especially the nature of the work—I mean, casual role—it was 2 days and 5 hours each, but it wasn't enough to sustain myself” (Mandeepa). These financial limitations meant that most participants living in a low socioeconomic area within Australia experienced food-related concerns and food insecurity. One participant stated “Then, with groceries, I’m budgeting it every week. I’m just supposed to be buying less than $50” (Kailani). Participants who experienced food insecurity during the pandemic expressed feelings of helplessness and substantial stress, often having to ration food or skip meals as a coping strategy, with Karlee explaining her challenges: “I would say financially, I already mentioned that, but that was a challenge, and then I think not having money to get food—I would try to eat one meal a day” (Karlee). Other participants in the low socioeconomic areas described having to borrow money from friends or family to purchase food: “Mostly it’s financially, because I ran out of my savings, and I contacted my mum and my sister. My sister sent me extra money to help buy food” (Kailani). While most participants in low socioeconomic areas reflected on increased hardship in relation to food access, one participant who lived in a low socioeconomic area noted that the pandemic changed their approach to managing meals for the better: “We've changed some of our purchasing habits. Even just that short experience of standard work from home lockdown has changed a lot of—we don’t buy as many meals out anymore. For lunches, there's a lot more packed stuff that we make or leftovers from meals” (Joshua).

### Reliance on food banks

The use of food banks and other non-governmental organisations to access a non-perishable food supply was “a service availed quite frequently” during the pandemic, most prominently among those participants from low socioeconomic areas. Such safety nets ensured a consistent food supply for those most in need, with one participant saying, “every week there were cartons of non-perishables and some fresh produce that were actually delivered to me, to be shared with my partner, which was really helpful” (Mandeepa). Another participant described that they were experiencing financial difficulties and were unable to afford food; she explained: “For me, I just wanted money for food I thought, so that's why I'd just go to the food bank” (Haimi) and was pleased that this service was available. Being able to seek the assistance of the food banks alleviated some of the food worries and insecurity experienced by participants, with one participant appreciative that they could: “I go to the free food places, just to top up my cupboards” (Dominic).

Cultural and religious groups also coordinated the delivery of foods to those within their communities that they knew were food-insecure, dropping the food in boxes at their doorstep: “They [Filipino groups] sometimes give a freshly caught fish, rice, bread, pasta sauce, corned beef, canned goods and everything” (Kailani). Kailani voiced that the food delivered by the cultural groups saved them at times: “If we don’t have anything to put on the table, we’ll just grab the corn beef.” Other participants discussed a sense of community when it came to food relief and support, saying, “The community came together. They were doing shopping for elderly and vulnerable people in my building, and they would be ringing me or cooking for me, those sorts of things” (Manaia). Emergency food relief came in all forms, including grocery gift cards, free meals and non-perishable food supplies; these were most often supplied by non-governmental organisations and religious groups.

However, reliance on food banks for access to a healthy food supply was relatively scarce among our sample, with most only being able to provide non-perishable items, meaning a lot of the food was of poor dietary quality. Participants described this as “so depressing”, with one communicating that “Just looking at that made me really sad. But yeah, that was a challenge” (Haimi); however, they were grateful for the support, as without it they were unsure of how they would manage.

### Stockpiling of food supplies

In contrast to participants living in low socioeconomic areas, participants in our sample within higher socioeconomic areas expressed dissimilar food concerns and were largely distressed by the inconvenience of their usual supplies not being available. To remedy this, many decided to support local hospitality businesses and ate “lots of take out. Lots of opulent food. Like decadent food.” (Sergio). While others in high socioeconomic areas had no concerns with food supply, one participant said, “Getting food was not an issue, no, no not really at all. We're quite lucky in that we have a—we're financially, maybe not rich, but we're not that precarious, and we live right next door to a supermarket, so even during lockdown, we'd make it a regular part of our routine to walk the dogs down and buy our groceries. Yeah, food was not an issue” (Trey). Another had a similar experience, saying, “We could get everything that we needed. There was no shortage there where we lived. In fact, it's amazing to think that Woolworths or Coles can get toilet paper at the present time” (Aaron). The majority of those within high socioeconomic areas were able to afford to stockpile food supplies, with one participant communicating, “So we were always stocked up with our vegetables and things like that. We did go out and buy heaps of stuff, although the pasta—limiting to pasta and things like that, which with only the three of us, we were doing okay, but my sister's got four kids, so they were struggling a little bit with the limits of what they could buy” (Alicia).

In comparison, those from lower socioeconomic areas did not have the financial ability to stockpile food: “We didn't stockpile, no” (Mandeepa), and another participant had a similar experience stating, “everyone was stocking up on toilet paper and food and everything, and we didn't have much in our pantry” (Haimi). However, for those who lived outside of urban areas and in agriculture-rich regions, fruit and vegetable markets continued to operate, meaning there was an abundant supply of fresh produce. One participant explained, “I mean the supermarkets ran out of the strangest things, but yeah, I mean, one of the benefits of living out of town is you tend to have a lot of dry stores, so that was fine, and since fresh vegetables—I mean we’ve got a lot of agriculture around us, so you get vegetable stalls and they continued to operate and never ran out” (Clara).

## Housing outcomes

Hand in hand with the burden of food security, many participants in our sample from low socioeconomic areas expressed emotional distress in relation to securing and maintaining adequate housing.

### Precariously housed

Many participants from low socioeconomic areas experienced feelings of helplessness and loss of control due to their insecure housing tenure. Those who described themselves as being precariously housed were almost exclusively females who lived in low socioeconomic areas. One participant talked about being verbally abused by her landlord and living in fear: “I was in a shared house where my landlord was very abusive verbally, and just it was terrible. At the time I didn't know about my rights, so I was constantly scared that I would—even though that couldn't happen, and I read about that in the news as well, but I was scared that I'd be kicked out of the house. So I wouldn't say anything and I was just in this house where there—you know, it was just a difficult situation. It was quite abusive and because of COVID, I couldn't really go out much, so I was just stuck in that space” (Haimi). Additionally, the landlord continually threatened to increase the rent, but without any employment, the participant felt she had to continue living in this threatening environment.

While prior to the pandemic there was a strain on housing within Australia, this was precipitated during the pandemic. In our sample, participants discussed the challenges with one lease ending and trying to find another available private rental or share house, describing it as very uprooting and increasing their anxiety levels saying, “From there, the lease ran out, which was when I moved to the house-sitting place, there was a period of time that was not covered between them, so it was about 3 weeks that I had to find a place to live. It was—the level of anxiety!” (Mandeepa). One participant described having to move “about four times” during the pandemic due to insecure housing. Conversely, a participant who resided in her own home before the pandemic was able to lease her house out and move in with her mother when she lost her employment during the first wave of the pandemic: “So in the end what I had to do was move in with my mum and rent out my house. Just so that—meet the mortgage” (Clara). Kailani spoke about moving to a rural town to commence a new job and the difficulty she experienced in finding housing. She did not have enough money to secure a short-term rental through Airbnb, and she ended up having to find housing in the next town. This made further challenges for Kailani in terms of transport to work, as she didn’t have a car: “I found a place, but then I always take the bus every day for 6 months, I think, I was taking the bus. Where the bus ride, in the morning and the afternoon, only goes once. So, if I’m going to miss it, I won’t be able to go back home or go to work. Then in the afternoon, I’m waiting for 2 hours for the bus so that I’ll go back—when I go back home” (Kailani). Furthermore, she also lived in fear in the shared house because she had to share with four males, which made her very uncomfortable and lonely, never having had to share with males previously. “Losing a sense of control” described the housing situation of most participants in our sample living in low socioeconomic areas.

### Housing stability

The ability to have stable and affordable housing was an experience that most participants who lived in high socioeconomic areas were fortunate enough to achieve. This was often associated with either having secure employment or owning their own home. Interestingly, housing stability was more evident among males in high socioeconomic areas. Despite Trey losing income during the pandemic, he talked about being lucky to have secure housing: “I have secure housing. My partner owns this house, which was a significant stress off our shoulders” (Trey). Similarly, another participant, Nick was able to gain secondary employment, enabling him to keep up with his mortgage payments: “I was able to maintain my mortgage payments on the house, so, no, it had no impact at all, the housing” (Nick). Having secure full-time employment and financial stability also assisted in housing security, with one participant who had recently moved out of her family home into a rental property explaining, “I’m being good to pay rent, I can buy things I wanted to buy, so I think it’s okay, and I’ve got a full-time job as well, so yeah, it’s okay” (Xiuying). Residing in a high socioeconomic area and living in a rental property, Sergio entertained the idea of asking for a rent reduction, but felt it would not be in his favour, saying, “So the landlord could turn around and go no and actually move out because it sounds like you might be a financial liability to me. So we didn’t ask for rent reduction. We still managed to pay rent, and we were never in a situation of housing precarity. So that was also fortunate” (Sergio). For some participants who had paid off their mortgage, housing was never an issue. One participant explained that “Not long ago I managed to finalise my mortgage, so yes, I’m very lucky with that” (Marcel), while another stated, “I’d been very fortunate, yeah, in that sense I’m a carer for my 92-year-old mother. So I don’t live in the same house but I live on the same property, which we own” (Parrie). Similarly, for participants, such as Aaron, who were self-funded retirees, housing stability was never a challenge, as he was fortunate enough to remain in his own home.

Although the majority of low-income participants were concerned about their housing stability, some were able to capitalise on a range of opportunities to secure their housing. One participant felt lucky to have a considerate landlord who assisted her substantially, meaning she did not have a pay rent for a period of time: “So instead of paying rent, I was able to help him with a couple of his other units because he had people move out. So I helped get them into condition for sale. So for doing that he gave me 4 months' rent free”(Manaia). Although Jayda lost employment during the pandemic, she was still about to negotiate paying her mortgage, meaning she had housing stability: “I’m buying my home so I was—I’m fine [inaudible]. Fortunately, I have my own home. So yeah, just paying my mortgage” (Jayda). Despite residing in a low socioeconomic area, participants who had existing social housing provisions in place were able to maintain their housing security. This was the case for Nyah who explained, “I mean because of the disability, I’m lucky to have housing commission housing, so there was not change for me” (Nyah).

## Psychological and emotional impact

The direct and indirect impacts on participants' psychological and emotional well-being during the COVID-19 pandemic varied substantially among those who lived in low socioeconomic areas compared to high socioeconomic areas. Participants in all socioeconomic areas were psychologically or emotionally impacted during the pandemic, however, this impact was often experienced more by those living in low socioeconomic areas.

### Well-being and quality of life

The many challenges faced by participants who resided within low socioeconomic areas during the pandemic were related not only to the uncertainty of the pandemic, but also dealing with social determinants of health that were exacerbated during the COVID-19 pandemic. Challenges such as loss of employment, lack of available finances, difficulties in housing stability, and issues with food security only worsened the situation for many and directly affected their well-being. One participant voiced, “My mental health suffered. I didn't think—I had never experienced depression before, not that I noticed anyway, but I went into a really, really dark place when I didn't know how I was going to pay bills and those sorts of things. Before I let people know my situation, things just got really dark and it was very easy to isolate so that people didn't know” (Manaia). This experience was after she had lost her employment, as well as recently losing her partner in an unexpected death. Manaia expressed a deep sense of loss and fear of losing the private rental she had shared with her partner that had memories for her. Additionally, being a New Zealand citizen, Manaia was ineligible for Australian Government financial assistance, which meant that options to address her precarious economic situation were limited.

Well-being and quality of life for most participants in our sample were referred to in terms of their mental status and to a lesser degree about exercise and healthy eating. One international student explained, “I think my quality of life was not the best. It was quite a mess, very stressful time. But I think over just amplified that by [a lot], so I think it was really tricky. There were a lot of things I didn't know and I think that just made it more stressful with COVID as well; not eating well, no money, no job, so I never really felt too good, so yeah, I had multiple deficiencies. I wasn't feeling good and I think that made it worse as well” (Haimi). Similarly, Kailani’s experience during the pandemic was worsened by her existing vulnerability and lack of financial and housing stability, affecting her quality of life and well-being substantially. Kailani shared, “So, a lot of anxieties have been the time—a lot of crying, too, during the night. It’s just my boyfriend who know about it. But I felt like I’m having—I don’t know, it’s like a lot of struggles. Inside I’m struggling. Waking up in the morning, I just feel like I just want to cry. I’m always thinking about the financial aspect, too. So, a lot of things are happening” (Kailani).

Indeed, for participants who already experienced mental health issues, the pandemic took a particular toll on their ability to manage. One participant expressed, “I would just curl up in bed and not get out of bed and just watch TV. I didn't really have a sense of day or night. I'd sleep when I was tired and be awake when I wasn't, so that didn't really help my mental health. It comforted me through, but it didn't help me improve and get beyond mental health issues” (Reuben). Reuben also stated that he felt “like a zombie”, with a very limited ability to function. Others residing in low socioeconomic areas also described a lack of ability to cope generally, saying, “Inside I’m struggling” to express the impact of COVID-19 on mental health and well-being.

While most participants described feeling negative effects on their well-being and quality of life during the pandemic, participants who resided within the highest socioeconomic areas of Australia discussed feeling less affected. Participants who expressed these sentiments had no changes in employment, were financially stable and thrived during the pandemic. A female participant stated, “Like, if anything, everyone was saying 2020 was such a shit year, but for us it was great. I was pregnant, so I loved working from home. I was probably really healthy, because I wasn't eating out or we weren't spending money and it was—I was actually sleeping—getting a lot of sleep and all that important stuff. Then obviously we had our baby, so for us 2020 was a wonderful year” (Alicia). Another male participant, Marcel, expressed a similar experience in that he felt the benefits of being about to work from home and the ability to continue his exercise regime, which meant his quality of life was not affected and he enjoyed the experience of lockdowns, saying, “I think overall I've coped very well with the situation. I don’t think there's been any real challenges to me personally. […] I did maintain an exercise regime through much of the working—the lockdown period. We were doing a lot of walking and stuff, so physical fitness was good” (Marcel).

## Discussion

This study sought to investigate the experiences of Australian adults concerning the impacts of the COVID-19 pandemic on the social determinants of health, while exploring whether this impacted their well-being. This study identified themes that were of relevance for participants living in low socioeconomic areas, including food-related concerns, precarious housing situations and the impacts that these had on their psychological and emotional well-being. Furthermore, this study recognised that there was a clear divide between the experiences of those living in low socioeconomic areas compared with adults living in high socioeconomic areas, with participants in low socioeconomic areas fairing worse in terms of exacerbated social determinants of health and consequent impacts on well-being. This was noticeably apparent when it came to food supply and housing stability, which are critical social determinants of health. Interconnected as basic human needs, food and housing are the prerequisites for health and well-being (Kirkpatrick and Tarasuk 2011). Those that live in poverty are also likely to experience both housing and food insecurity, reflecting the impacts of financial constraints. Having to choose between paying rent or paying for food is the reality for low-income households; however, if they were provided with affordable housing options, then these households would have greater income to purchase food (Arshad 2022). It has been well documented in the literature that low-income families spend a considerable amount of their income on securing housing, so as the cost for housing increases so does food insecurity (Fafard St-Germain and Tarasuk 2020; Kirkpatrick and Tarasuk 2011). For the most part, a disadvantaged social status has made the impacts of COVID-19 worse for many Australians, with those already socially and economically vulnerable disproportionately affected by the pandemic.

Food insecurity, defined as the inability to acquire adequate food supply (McKay et al. 2019), was experienced by the majority of participants who were from low socioeconomic areas in this study. According to the WHO Commission on Social Determinants of Health (CSDH) framework, social, economic and political mechanisms define socioeconomic positions based on income, education, gender, ethnicity and occupation. socioeconomic positions then shape how people experience differences in vulnerability to illness and in exposure to a public health crisis (Palmer et al. 2019) such as a pandemic. This explains how those participants from a higher socioeconomic area may lose their employment during the pandemic but remain food-secure. Where on the other hand, participants from low socioeconomic areas who lost their employment became food-insecure or experienced worsened food security. While food security may have been a challenge for some of the participants within low socioeconomic areas prior to the pandemic, the pandemic has amplified this social determinant of health for these participants. The experience for the participants in this study is that they had to seek food assistance at food banks and through non-governmental organisations and cultural groups, with some participants skipping meals and rationing their food supply. Lack of access to an adequate food supply, even if temporary, is associated with poor nutritional intake and can impact long-term health (Kent et al. 2020). Aligning with the literature, participants’ experience of reliance on the food banks as a food source in this study highlights the poor dietary quality. Food security enables optimal physical health and well-being (Pooler et al. 2019); without this, individuals may suffer from ill health, having an impact on their quality of life and overall well-being.

Social determinants of health do not exist independently from one another, as there are an abundance of factors involved, and the inequalities between socioeconomic groups arise in response to a range of unequal opportunities, unequal conditions and unequal resources (Lundberg 2020). That is, people can be affected by a collective of social determinants of health, such as food insecurity, gender, ethnicity, education and housing instability, as they often coincide (Thornton and Persaud 2018). This was a key element of this participant experience in this study. This is portrayed in the case study of Mandeepa (Fig. 2), whereby her food and housing insecurity is impacted by other existing social determinants of health and together influencing her total well-being. Pre-pandemic, food insecurity within Australia was estimated to be between 5.1% and 10.6% (McKay et al. 2019); however, our previous research shows that this increased to 22% during the pandemic (Green et al. 2022). The increase in food insecurity during the pandemic is an accumulation of social and economic disadvantage experienced by adults, particularly within low socioeconomic areas.

Many participants experienced loss of employment and loss of income during the pandemic that made them economically vulnerable and ultimately food-insecure. It is important to note that participants in this study that expressed food-related concerns were predominately female and from migrant communities. This is consistent with the global literature that demonstrates that women are more likely to report food insecurity, although there is limited evidence of the reasons for the gender difference (Carter et al. 2010; Maynard et al. 2018). One theory is that women are perhaps more likely to be sole parents, may be less educated and live in poverty compared to males (Grimaccia and Naccarato 2020). Additionally, there is a direct association with low income and food insecurity, with a study conducted in New Zealand reporting that more women are in low-income households than males, with a relationship between low-income households, social welfare and access to food banks (Carter et al. 2010).

The supply and demand for housing during the pandemic has uncovered the fundamental weaknesses within the Australian housing system (Buckle et al. 2020). In this study, participants who lived in low socioeconomic areas experienced precarious housing, describing the impact that having to move multiple times during the pandemic had on their well-being. Participants also expressed their experiences of trying to secure housing in a regional area of Australia as challenging and losing a sense of control with lack of supply and lack of finances to be able to secure even short-term housing. While the Australian government initiated a residential tenancy support package in the early stages of the pandemic to protect tenants against eviction if they were unable to meet their rental payments (New South Wales Government Fair Trading 2021), there was no deliberate action to increase housing availability and affordability for those with financial pressures or those experiencing loss of employment or income due to the pandemic. Australia has a chronic housing shortage, predominately affordable and secure housing, with the pandemic amplifying and bringing this issue to the forefront (Buckle et al. 2020). The ability to work from home during the pandemic increased the demand for housing in regional areas within Australia, as the ability to work remotely no longer dictated that people live in metropolitan areas (Regional Australia Institute 2022). Therefore, people with high incomes and immediate resources chose to occupy regional and rural locations, which in turn decreased the availability of housing for people who were already living in these regional areas.

This study has highlighted that housing instability was mostly the experience of women, rather than men. Australia’s neoliberal preference for a private rental market has led to a lack of affordable housing options and shrinking social housing provision, leaving many women homeless (Blunden and Flanagan 2020). This is likely to have been magnified during the pandemic, with limited housing availability and financial pressures further exacerbating relationship stress (Green et al. 2021).

The housing- and food-related stresses experienced by participants in this study has influenced their overall well-being, with many discussing the negative effect they had on their mental health, creating or worsening anxiety and depression. This is consistent with the findings of quantitative studies demonstrating that as food insecurity worsens, well-being deteriorates, and when food insecurity is apparent it is associated with depression, stress, and anxiety (Seivwright et al. 2020). Similarly, there is a bi-directional relationship between housing instability and homelessness and well-being, whereby stress, created by housing instability, can weaken an individual’s capacity to cope, affecting their overall well-being (Padgett 2020). Furthermore, it is evident from this study that women’s well-being was substantially impacted when compared to men. This is not unexpected, given that many women experienced either food insecurity and/or housing instability.

It is evident from this research that there exists a socioeconomic status variation on the effects on Australians’ well-being during the pandemic based on their existing or amplified experiences of the social determinants of health. Recommendations for public health in addressing the social determinants of health include lobbying governments to incorporate the social determinants of health in all policies, implementing strategies to address food security, and implementing public health interventions to address housing affordability.

## Strengths and limitations

A strength of this study is that the data were collected via purposively selected participants that allowed for a diverse sample from various socioeconomic areas and geographical locations. Using qualitative methods allowed participants lived experiences to be highlighted, which was particularly important given that this is one of the few studies that have explored participants lived experiences of the social determinants of health during the COVID-19 pandemic. While every attempt was made to conduct all interviews through videoconference, three interviews had to be conducted over the phone, which meant body language and eye contact were not visible and may have impacted on the quality and interpretation of the data (Irvine et al. 2013). This was however mitigated through careful listening, which enabled the researcher to note change in voice tone, or rapid speech, and to replace nods and facial expressions that would normally demonstrate interest with verbal signals. It must be acknowledged that there are disadvantages of using a purposive sampling methodology, as it is subject to researcher bias. Additionally, in terms of generalisation, this qualitative research was not designed to provide opportunity for generalisation but rather to provide perspective and understanding of the issues in a given sample. Generalization is not possible in this study because the sample is limited by purposive sampling of participants, and the extent to which the findings are relevant to other settings and populations is undetermined. However, using a diverse sample encouraged discussion of a wide and varying experience, rather than from those with one participant characteristic, such as gender or high socioeconomic status. It must also be noted that not all participants were Australian citizens, which may affect the experiences and results of this study, with international students not receiving the same support from the Australian government that Australian citizens would receive.

## Conclusion

This study highlights the social and economic divide of the COVID-19 pandemic experience and impacts. The pandemic has amplified existing social determinants of health experienced by those within low socioeconomic areas, particularly those who are female and from migrant communities, demonstrating that social and health inequalities are shaped by the conditions in which people are born, live and work. Overall, the well-being of participants from low socioeconomic areas decreased in response to their experiences and challenges with food insecurity and housing instability, highlighting the need for housing affordability strategies and funding of emergency food relief initiatives. Food access, insecurity and availability for local communities, particularly for those in areas with high socioeconomic disadvantage, can be improved to address some of the barriers associated with food security through providing café/supermarket meal vouchers, access to community gardens and school food programmes. Housing affordability projects require programme expansion and capacity in terms of availability, including an increase in supply of social and public housing. Additionally, there needs to be an increase in rental assistance provided to people within lower socioeconomic areas, especially those in the private rental market, to ensure they have access to affordable housing.

## Data Availability

Data are available at the request of the corresponding author.
